# Tree height explains mortality risk during an intense drought

**DOI:** 10.1038/s41467-019-12380-6

**Published:** 2019-09-26

**Authors:** Atticus E. L. Stovall, Herman Shugart, Xi Yang

**Affiliations:** 1 NASA Goddard Space Flight Center, 8800 Greenbelt Rd., Greenbelt, MD USA; 2 Department of Environmental Sciences, University of Virginia, 291 McCormick Rd., Charlottesville, VA USA

**Keywords:** Climate-change ecology, Ecophysiology, Forest ecology

## Abstract

Forest mortality is accelerating due to climate change and the largest trees may be at the greatest risk, threatening critical ecological, economic, and social benefits. Here, we combine high-resolution airborne LiDAR and optical data to track tree-level mortality rates for ~2 million trees in California over 8 years, showing that tree height is the strongest predictor of mortality during extreme drought. Large trees die at twice the rate of small trees and environmental gradients of temperature, water, and competition control the intensity of the height-mortality relationship. These findings suggest that future persistent drought may cause widespread mortality of the largest trees on Earth.

## Abstract (web summary)

Drought is intensifying due to climate change, impacting forests globally. Here, the authors track nearly 2 million trees through severe drought and show that tree height is the greatest predictor of mortality risk, suggesting that the tallest trees may be the most vulnerable.

## Introduction

Drought is likely to become more widespread, prolonged, and extreme over the next century^1^. Tropical^2,3^, temperate^4^, and boreal^5^ forests are already experiencing historically unprecedented drought accompanied with massive increases in tree mortality^6^. Severe drought conditions strongly increase ambient temperature and decrease precipitation, which in concert push trees to their physiological limits^7^ by increasing vapor pressure deficit (VPD)^8^. Widespread tree death changes ecosystem structure and produces consequent feedbacks in surface energy balance, which reduces the capacity of forests to mediate climate and exacerbates drought conditions^9^. Large trees contribute to the most substantial tree-related micro-climatological benefits^10^—highlighting the necessity for constraining mortality expectations of these trees to better predict future vegetation-climate feedbacks.

Extreme drought may intensify height-dependent tree mortality^11^, drastically shifting future ecosystem structure and demographics. The main mechanisms of tree death during drought are carbon starvation and hydraulic failure^12^—both of which are exacerbated by the presence of biotic agents^13^ (e.g., beetles). Theoretical^14^ and empirical^11,15,16^ work indicates an increased risk for large trees under drought stress. However, higher mortality in small trees is reported, as well^4,5,17,18^. This disagreement in past mortality studies highlights the system-dependent response of trees during drought. Past work has relied on global aggregation of plot-level datasets to compile sufficient observations for a confident assessment of risk factors^11,19^, but these syntheses are across past observations, which are limited in their extent and/or sample size. Spatially extensive tree-level assessments in specific forest types are needed to substantially improve future predictions of global tree mortality^20^.

To this end, we track the mortality of 1.8 million trees in conifer-dominated forests over 8 years during one of the most severe and prolonged droughts recorded in the Southwest of North America to determine the primary cause of tree death. We combine high-resolution (sub-meter) airborne three-dimensional and optical data for over 40,000 ha within the southern Sierra Nevada forest to locate individual trees and assess mortality across the landscape. We identify and track crown-level tree mortality (88% accuracy, Supplementary Figs. 1–4) in the high-resolution airborne imagery for each acquisition year and, during the highest mortality period (2014–2016), test biological, topographic, soil, and climatological variables as drivers of tree-level mortality probability and mortality rate. We find that tree height is the single most important predictor of tree death during drought. Nearly half of all trees >30 m tall died during the study period—a rate more than double that of trees <15 m tall. We then show how extremes in the environment mediate or exacerbate mortality intensity. As environmental stressors increase, we find large trees are non-linearly impacted, suggesting more frequent and extreme drought may be most detrimental to the largest trees on Earth.

## Results

### Temporal mortality trends

**Fig. 1:**
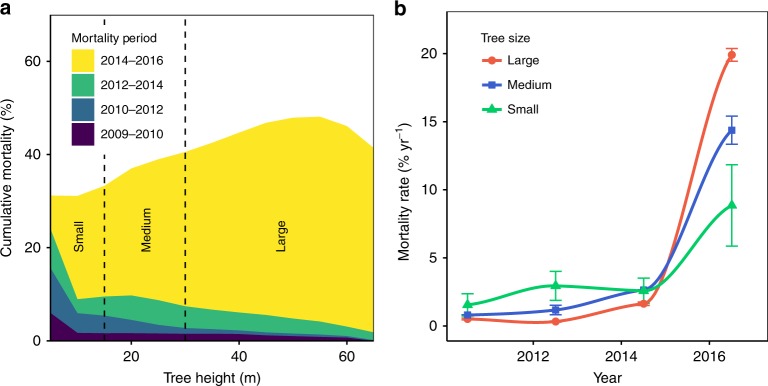
**a** Height-dependent cumulative mortality and **b** trend in mortality rate of small (< 15 m), medium (15–30 m), and large (> 30 m) trees since the start of drought. Our temporal analysis shows 740,724 trees died in the 40,854 ha study area over an eight-year period (41% mortality). **a** The cumulative mortality of all years reflects the relative percent mortality of all trees in 5 m height classes (e.g., at the study’s end, 40% of all 40 m trees died). Vertical lines show the range of tree heights defined as small, medium, and large. Prior to the start of drought, all tree heights have similar mortality rates, with elevated mortality in smaller tree populations. As drought persists, the mortality increased in small trees, while it remained lower in large trees. At the late stages of drought, the mortality rate of all trees increases, with large trees surpassing the rates of smaller tree heights, and becoming the most vulnerable population. The error bars indicate 95% confidence of mean mortality rate within height class

The temporal trend of tree mortality clearly shows height-dependent trajectories (Fig. 1). We initially tested the relative percent mortality at 5 m tree height classes from 2009 to 2016 to determine the presence of a height-dependent relationship over the drought period (Fig. 1a). We simplify the analysis into small (< 15 m), medium (15–30 m), and large (> 30 m) height classes for interpretation of the time series (Fig. 1b). In total, we identified 305,600 small trees, 855,730 medium trees, and 647,004 large trees. From 2009 to 2010, mortality was 1.56, 0.80, and 0.58% yr^−1^ for small, medium, and large trees, respectively. From 2010 to 2014, mortality rate rose across all tree heights and by 2014 cumulative mortality was 14.15%, 9.24%, and 5.09% for small, medium, and large trees, respectively. In 2016, large-tree mortality rate surged from the background rate of ~1–19.90% yr^−1^, while small and medium height trees died at a rate of 8.85 and 14.38% yr^−1^. By 2016, cumulative mortality was highest in large trees (45%), followed by medium (38%) and small (32%) trees. Overall, we estimate that 740,724 or 41% of the measured trees died from 2009 to 2016.

### Mortality drivers

**Fig. 2:**
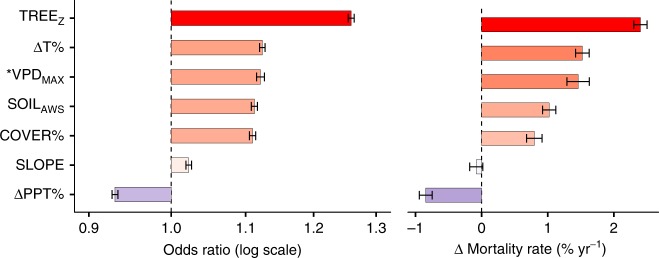
Tree height is the dominant controlling factor of tree death during drought, followed by temperature, vapor pressure deficit, available water storage, tree cover, and precipitation. Tree height (TREE_Z_), tree cover (COVER%), maximum vapor pressure deficit (VPD_MAX_), temperature (ΔT%), water (ΔPPT%), slope, and soil texture (SOIL_AWC_) explain the **a** Odds of tree death and [b] change in mortality rate (*n* = 1,808,334). Variables with odds ratios greater than one (vertical dashed line) increase mortality risk (red) and less than one decrease mortality risk (blue). Error bars represent the 95% confidence in the estimates of the odds ratio and mortality rate. *Note: VPD_MAX_ is highly correlated with ΔT% and ΔPPT%, so we derive odds ratio and change in mortality rate for this variable in a reduced model (see Supplementary Fig. 5), excluding temperature and precipitation

Tree height was the strongest predictor of tree mortality, compared with maximum vapor pressure deficit, maximum temperature, precipitation, available water storage, cover, and slope (Fig. 2a, b). For every 10 m taller a tree grows, the risk of mortality increases by 1.26 times (95% CI 1.254–1.264) and mortality rate increases by 2.40% yr^−1^ (95% CI 2.3–2.5). Vapor pressure deficit is a primary predictor of tree mortality, but is highly correlated with temperature and precipitation (*r* = 0.98 and 0.96, respectively). To test the strength of vapor pressure deficit in predicting tree mortality we ran a reduced model, substituting vapor pressure deficit for temperature and precipitation. We found a 0.4 kPa increase in maximum VPD increases mortality risk by 1.12 times (95% CI 1.116–1.127) and mortality rate increases 1.46% yr^−1^ (95% CI 1.29–1.63; Supplementary Fig. 5). Tree height is even more important in the reduced VPD model, with an increase in overall mortality risk to 1.35 times (95% CI 1.340–1.350) and mortality rate to 3.42% yr^−1^ (95% CI 3.28–3.56). Temperature and precipitation, like VPD, are strong drivers of tree mortality. We found that for every 2.9% (0.4 °C) increase in average maximum temperature during the study period above historical levels results in an increase in tree mortality risk by 1.123 times (95% CI 1.120–1.128) and rate by 1.52% yr^−1^ (95% CI 1.42–1.62). Every decrease in precipitation by 4% (35 mm) below historical averages increases mortality risk by 1.070 times (95% CI 1.066–1.073) and rate by 0.85% yr^−1^ (95% CI 0.75–0.94). Increasing soil water availability by 6.2 mm led to 1.112 times (95% CI 1.108–1.117) higher risk of mortality and a 1% yr^−1^ (95% CI 0.92–1.12) increase in mortality rate. Finally, increasing tree cover by 17% increased mortality risk by 1.110 times (95% CI 1.105–1.114) and mortality rate by 0.80% yr^−1^ (95% CI 0.68–0.91).

**Fig. 3:**
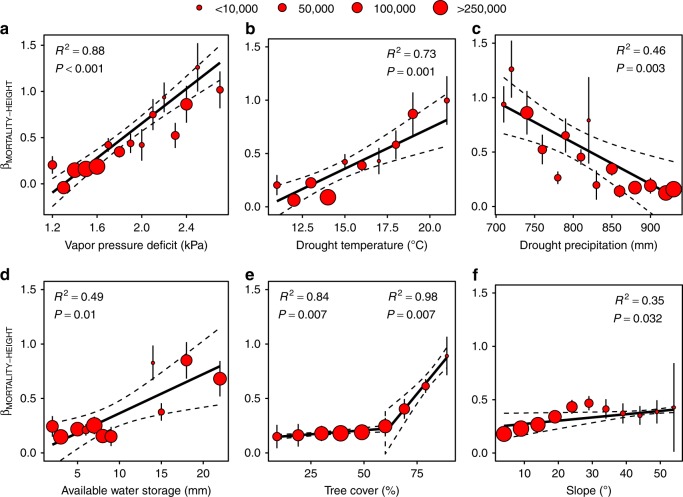
Environmental gradients drive the susceptibility of large trees during drought (*β*_MORTALITY-HEIGHT_ > 0). Higher vapor pressure deficit **a** during drought—resulting from higher temperatures **b** and lower precipitation **c**—disproportionately increase mortality in large trees. **d** Large trees adapted to soils with higher available water storage are at increased risk during drought. **e** Areas with >50% tree cover have elevated large-tree mortality rate. **f** Large trees are less likely to survive on steeply sloped areas during drought. Regression slopes and *R*^2^ values are weighted by 1/*σ*^2^ and point size reflects number of tree observations. Error bars represent the 95% confidence in the slope of the mortality-height relationship (*β*_MORTALITY-HEIGHT_). See Supplementary Table 2 for slope and error coefficients

Biological and environmental variables modulate the relationship between tree mortality and height. The slope of the mortality-height relationship (*β*_MORTALITY-HEIGHT_) varies linearly with nearly every environmental variable tested (Fig. 3; Supplementary Table 2). Of the variables tested, maximum VPD (*β*_1_ = 0.0938% yr^−1^ m^−1^ kPa^−1^, *R*^2^ = 0.88) and maximum temperature (*β*_1_ = 0.0762% yr^−1^ m^−1^ °C^−1^, *R*^2^ = 0.73) most strongly increased intensity of the height-mortality relationship. Under more mild conditions (e.g., low VPD) *β*_MORTALITY-HEIGHT_ remains close to 0, indicating a lack of height-dependent mortality relationship. *β*_MORTALITY-HEIGHT_ increases linearly as conditions move to the opposite extreme (e.g., high VPD). For instance, trees that experienced an average maximum vapor pressure deficit greater than 2.4 kPa during the study period had an average mortality rate of 1.05% yr^−1^ for each additional meter taller the trees grew. In areas experiencing these levels of high maximum VPD, the mortality rate for a 10 m tall tree was 6% yr^−1^, while trees above 30 m were lost at ~30% yr^−1^ (5.3 times greater). We found similar, albeit weaker, linear relationships for precipitation (*β*_1_ = −0.0038% yr^−1^ m^−1^ mm^−1^, *R*^2^ = 0.46), available water storage (*β*_1_ = 0.0938% yr^−1^ m^−1^ mm^−1^, *R*^2^ = 0.49), and slope (*β*_1_ = 0.0030% yr^−1^ m^−1^ %^−1^, *R*^2^ = 0.35). Tree cover did not have a clear effect below 50% (*β*_1_ = 0.1481% yr^−1^ m^−1^ %^−1^, *R*^2^ = 0.83), but above 50% the height-mortality relationship was strong (*β*_1_ = 2.371% yr^−1^ m^−1^  %^−1^, *R*^2^ = 0.98).

## Discussion

Over the drought period, large trees become more vulnerable than small trees (Fig. 1a, b). Initially we observe low mortality rate (1–5%), with slightly higher rates in small trees (5–10 m), reflecting typical competition-driven background mortality rates in forest ecosystems. The peak in small-tree mortality early in the drought suggests shorter trees may be more vulnerable under shorter duration and intense droughts. However, this would need to be demonstrated in additional drought events of varying length and intensity. As drought persists, large-tree mortality is 1.5–2.7 times higher than medium- and small trees (contrary to earlier work in California^8^). We attribute this temporal differentiation in height-dependent mortality risk to the strong relationship between leaf area, water, and carbon requirements for sustained productivity. More persistent drought increases the risk of carbon starvation^13^, thus increasing mortality risk for large trees due to their relatively higher metabolic requirements^21^. Dense and shallow roots in small trees may also improve survival odds^13^ by enabling rapid water uptake when precipitation finally occurs. Given the dramatic increase in tree mortality from 2014 to 2016, we focus the remainder of our analysis on determining the primary cause of mortality during this period.

Tree height is the single most important predictor of both mortality risk and mortality rate during this drought (Fig. 2a, b). These findings support theoretical work that suggests the internal hydraulic structure of trees alone is a major driver of tree mortality risk^14^. The hydraulic structure of trees commands such strong control over mortality since effects of gravity and xylem-wall resistance increase non-linearly with tree height^14,22^. The greatest hydraulic safety margins are observed in Gymnosperms—the dominant tree group in this region. Conversely, Angiosperms^23^ have low hydraulic safety margins, making flowering trees more susceptible under drought stress. The extreme mortality rates observed here may be exceeded in flowering tree populations under a changing climate and prolonged future drought^23^, potentially shifting future forest species composition^24^. A notable exception is the *Pinus* genus, which has undergone widespread dieback^25^ and is common at the lower elevations in our study. Higher temperature and deficit in precipitation elevates vapor pressure deficit reducing tree survival across whole forest stands^8^, but our findings indicate climate is of less absolute importance compared with individual tree height, at least in this region (Fig. 2a, b).

Climatic gradients in the environment control large-tree mortality rate (Fig. 3a–c), which explains the contrary height-dependent drought risk seen across sites in global studies^11^. The observed lowest temperatures and highest precipitations produce a near zero slope in the mortality-height relationship (i.e., suppressing height-dependent mortality risk). As temperatures rise and precipitation drops, vapor pressure deficit increases^8^ and large trees are more detrimentally affected, indicated by a linear increase in the slope coefficient of the mortality-height model (*β*_1_ > 0). The environmental control of mortality is nonlinear (*M* = *β*_0_ + *β*_1_*Z*_TREE_Δ_ENV_, where *M* is mortality rate, *Z*_TREE_ is tree height, and Δ_ENV_ is changing environmental condition)—resulting in a more precipitous increase in risk for large trees with relatively small changes in climate. The climate-sensitive nature of large trees could increase their mortality risk in the face of a rapidly changing global climate and extreme weather events^14^.

Local adaptation to higher soil water availability increases risk of mortality in large trees (Fig. 3d), supporting previous work showing a direct relationship between xylem embolism resistance and the level of water stress experienced^13,23^. Essentially, the xylem structures of trees grown under favorable conditions are optimized for water flow, not drought resistance^13^. The tallest trees are also more likely to grow in deep, moisture- and nutrient-rich soils^26^, concentrating the largest individuals in locations of highest risk and increasing susceptibility during drought^27^.

Tall trees growing in forests with high percent cover (> 50%) were more likely to die during this extreme drought (Fig. 3e), specifically due to competition for water, light, and nutrients^28^. Survival in drought-stricken environments is a delicate balance between long-term competition^29^—encouraging rapid growth to capture light—and heightened risk from a longer root-to-shoot pathway^14^. The absence of a relationship below 50% is indicative of a competition-driven effect. Specifically, belowground competition for water is likely to be the primary driver of increased mortality in dry forests with high cover^30^. Our findings suggest the combined impact of competition and frequent drought, along with a clear height dependency of tree susceptibility, are likely to disproportionately impact large trees in the future.

Large trees hold half of all mature-forest carbon, globally^10^, and we expect changing climate to most directly impact these trees^11,14–16^. Worldwide loss of large trees would result in a significant terrestrial carbon flux to the atmosphere, further exacerbating global carbon emissions^31^. As more trees die, forest fuel-load increases, along with the risk of wildfire, accelerating loss of carbon from forests^7^ (e.g., 2018 fires in California, USA). Shifting climate and increasing disturbance can shift whole ecosystems to alternate states^9^. While our study is unable to conclusively infer trends in long-term vegetation shifts, increasing prevalence of drought may detrimentally affect large trees—reducing maximum potential carbon storage and carbon residence time^10^. Managing of forests to store and sequester carbon over the next century will require careful consideration of height-dependent risk factors.

Extreme drought is becoming more frequent^1^ and is already causing massive shifts in ecosystem structure and function^6,32^. Our findings suggest that large trees are more likely than small trees to succumb to extreme drought. The loss of large trees would disrupt essential local^9^ and global^10,31,32^ ecological, economic, and social benefits. In a relatively small area, our study covers a wide range of environmental conditions (Supplementary Table 1; Supplementary Fig. 6), so we evaluate the global range of forests with similar climate (see Methods). A total of 72, 42, and 14% of forests experience the range of maximum temperature, annual precipitation, and combination of both found in the current study. At least 14% of Earth’s conifer-containing forests fall within the climatic range observed in this study—suggesting our findings may be more broadly applicable. We expect death in large trees will be mediated by local variations in vapor pressure deficit, temperature, precipitation, adaptation, and competition. Specifically, dense forests^28^ with historically favorable conditions^23^ are considered extremely high-risk during drought and should be managed to reduce catastrophic mortality (e.g., stand thinning). Our work reconciles past, seemingly contrary, findings concerning height-dependent mortality risk—we clearly show how multiple environmental factors explain the wide variability in the strength of the mortality-height relationship. Our results provide landscape-scale explanation of tree-level function that will be key in predicting forest mortality, carbon storage potential, and vegetation-climate feedbacks in next-generation earth-system models.

## Methods

### Crown mortality classification and mapping

We mapped tree-level mortality and tree height using high-resolution airborne data. We used sub-meter (0.6–1 m) NAIP imagery—freely available annual or biennial flights during the growing season by USDA—to classify tree crowns as dead or alive. All imagery was corrected for atmospheric effects by the vendor prior to analysis. We acquired all available imagery at the three sites from one year prior to the California drought (2009) until the most recently available imagery (2016), for a total of 5 analysis years per site. We automatically classified the NAIP imagery as sunlit or shaded within dead or living forest areas using training data and maximum likelihood classification (ArcMap 10.5.1).

Canopy height was estimated with airborne LiDAR (Light Detection And Ranging) data collected in 2013 over the SOAP and TEAK sites within the National Ecological Observatory Network^33^. We estimate canopy height from two 3D models reconstructing the ground and canopy surface. By subtracting the ground elevation from the canopy surface, we estimated canopy height across the study area. The high-resolution (0.6 m) canopy height model provided detailed information on tree-level canopy dimensions that allowed us to separate the 3D model into individual tree crowns.

Tree crowns are detected with a local maxima approach (*lastrees* function in the lidR R package), relying on a moving window to determine local high points in the model of canopy height^34^. The identified high points serve as potential crown locations^35^. Crowns are segmented using small-scale watershed delineation^36^, by first inverting the surface model and finding the approximate edges of the “watershed”. The watershed approach is an established and reliable approach in the forest types included in this study since most trees are conical in shape with little overlap, facilitating location of crown tops and segmentation.

Tree-level crown mortality was based on a threshold of percent of crown death. We assigned all dead pixels in our classified imagery a value of 1 and all remaining pixels as 0. Thus, crown-level averages of theses pixels represent the percent of a single tree crown that is identified as dead. We manually validated our tree-level mortality estimates to determine the highest accuracy threshold at which to identify a tree crown as dead. Sensitivity to the threshold was tested from 0 to 1 and 0.375 or 37.5% crown death provided the highest unbiased (88%) classification accuracy (Supplementary Figs. 1–4). All subsequent analyses assumed crowns with >37.5% death were dead trees.

### Temporal analysis

Total mortality and mortality rate were calculated by first binning tree-level mortality estimates by 5 m height-class intervals. We estimated mortality rate by totaling the dead trees in each height bin and tracking individual crown status over the 8-year study period. Trees identified as dead were removed from subsequent analysis years so as not to inflate estimates of mortality. The mortality rate was calculated as the percent mortality (dead individuals divided by total number of trees) divided by the number of years since the last image acquisition. We classified tree height into three categories: small (5–15 m), medium (15–30 m), and large (> 30 m). Within each class, we tracked tree mortality rate across the two sites.

### Mortality driver analysis

Environmental gradients were tested as drivers in tree mortality in addition to tree height. We used WorldClim2.0 estimates of maximum temperature and annual precipitation based on climate normals^37^. We used PRISM estimates of vapor pressure deficit, temperature, and precipitation during the drought period from gridded weather data^33^. For all PRISM data, we averaged the yearly estimates over the study period for a single gridded estimate of maximum VPD, maximum temperature, and precipitation from 2009 to 2016. Available soil water storage estimates, a 150 cm profile-summed estimate of available water storage based on soil texture, were also retrieved for our analysis from the SSURGO database^38^. We calculated slope from the LiDAR-derived ground surface model. We also included an estimate of forest cover above 5 m in our analysis by aggregating a classified (greater or less than 5 m) LiDAR canopy height model to 1 ha resolution. All variables were matched to the ~2 million trees in our analysis. We checked variance inflation factors (VIFs) for our predictor variables in both statistical models to ensure minimal collinearity (VIF < 2). Temperature and precipitation were strongly collinear. We remedied collinearity by describing the two variables in terms of percent deviation from historical means—essentially the yearly relative climate anomaly. All variables in both of the final models had VIFs <2.

We tested the strength of environmental drivers in explaining mortality likelihood with bivariate logistic regression. Tree height, cover, and the environmental gradients were treated as predictors for mortality likelihood. We compared standardized coefficients in a multiple bivariate logistic regression model to determine variable importance. A single unit change in a standardized variable represents one standard deviation from the mean, so all variable coefficients can be directly compared. Variables that were not normally distributed (tree height and slope) were log transformed prior to analysis. We represent the coefficients of each variable in the model with odds ratio, indicating the relative increase or decrease in mortality likelihood for every subsequent change in standard deviation from the mean, while controlling for all other environmental variables. We observed <3% background mortality in this study prior to the drought, so we assumed the odds ratio is a close approximation of the relative risk in the study population.

We analyzed the drivers of mortality rate using multiple linear regression modeling. We included an identical set of predictors as the bivariate model, but replaced mortality status with mortality rate. We derived mortality rate with narrow bins across all environmental variables. For each corresponding set of unique bin combinations, we calculated the mortality rate as the number of dead trees divided by the total number of trees in the same bin, dividing by two to represent the 2-year time-lag between the 2010 and 2016 NAIP acquisitions. As such, mortality rate in this study was unable to reach above 50% for any time period, though this likely occurred in some areas. We used the same transformations and standardization as the bivariate logistic regression model to ensure consistency.

To test the impact of climate on the height-mortality relationship, we developed sample-size weighted linear relationships for each binned interval in the environmental data. The slope of the linear model described the impact of a particular environmental gradient on the intensity of the height-mortality relationship. We regressed the significant estimates of the slope of the height-mortality relationship against the corresponding environmental gradient, weighted by *σ*^−1^ of the regression slope, allowing us to determine how an increase (or decrease) in a particular environmental factor may impact the strength of the mortality-height relationship. The derived slope from our analysis essentially describes the impact of different environmental gradients on large trees, with higher values representing a strong mortality-height effect for a given set of environmental conditions.

### Global extent of forests with overlapping climate

We evaluate the global distribution of forests^39^ with a maximum temperature and annual precipitation that fall within the range observed in this study (Supplementary Table 1). Global maximum temperature and average precipitation are derived from WorldClim2.0 2.5 degree data^37^. Within this overlapping climatic^37^ range, we quantified the extent of conifer-containing forests using the World Wildlife Fund’s Terrestrial Ecoregions product^39,40^.

### Reporting summary

Further information on research design is available in the Nature Research Reporting Summary linked to this article.

## Supplementary information

Supplementary Information

Description of Additional Supplementary Files

Supplementary Movie 1

Reporting Summary

## Data Availability

The tree-level data that support the findings of this study are available through figshare with the identifier 10.6084/m9.figshare.7609193.v1^41^.

## Code availability

The analysis for this study is available on GitHub at https://github.com/aestovall/tree_mortality.
