# Supplementary material for: Tree height explains mortality risk during an intense drought
Source: Nat Commun. 2019 Sep 26;10:4385. doi: 10.1038/s41467-019-12380-6 (PMC6763443; doi:10.1038/s41467-019-12380-6)
Supplement: Supplementary file 1 — Supplementary Information [file 41467_2019_12380_MOESM1_ESM.pdf]

Supplementary Information

Title:
Tree height increases mortality risk during extreme drought

Authors:
Atticus Stovall[1,2*], Herman Shugart[2] & Xi Yang[2]

Author Affiliations:
[1]NASA Goddard Space Flight Center, 8800 Greenbelt Rd., Greenbelt, MD, United States
[2]Department of Environmental Sciences, University of Virginia, 291 McCormick Rd., Charlottesville, VA, United States

Corresponding Author:
Atticus Stovall, Ph.D.
8800 Greenbelt Rd., Building 33
Greenbelt, MD, 20771
Phone: 301.614.6677
Email: atticus.e.stovall@nasa.gov

*Corresponding Author

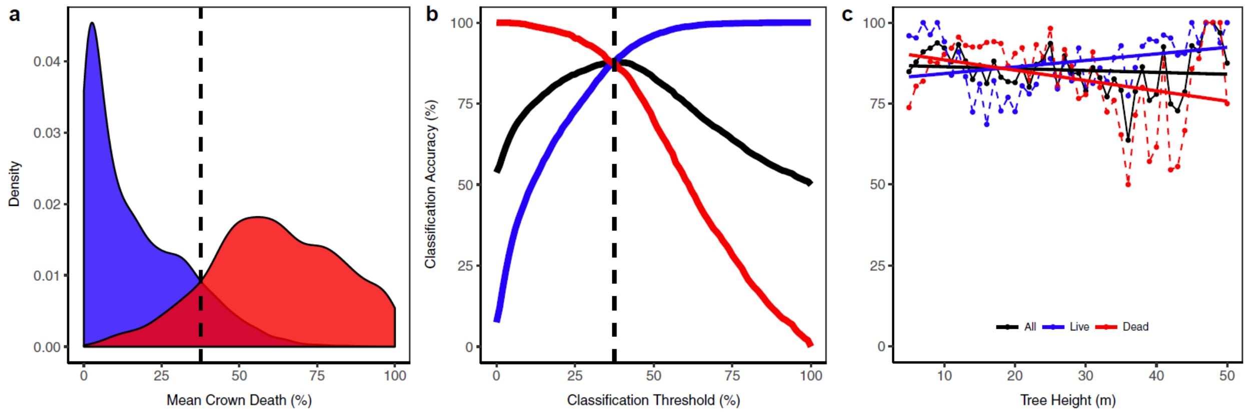

Supplementary Figure 1: Accuracy assessment of tree mortality detection (n = 5474). We used positively identified validation trees within the study area to assess error of the classification method. a) The percent of classified dead crown pixels allowed differentiation of dead and living trees. b) We assessed impact on classification error with varying classification threshold and determined 37.5% crown death was the most accurate and unbiased threshold for mortality detection. c) We assessed the relationship between overall (black), live (blue), and dead (red) classification accuracy with respect to height, to ensure a lack of size-dependency in our analysis. Dead tree classification had a near-zero slope, with a slight decrease in accuracy with height. Overall accuracy of the method was 88% (kappa = 0.7254). Live and dead tree detection accuracy was 88% and 87%, respectively.

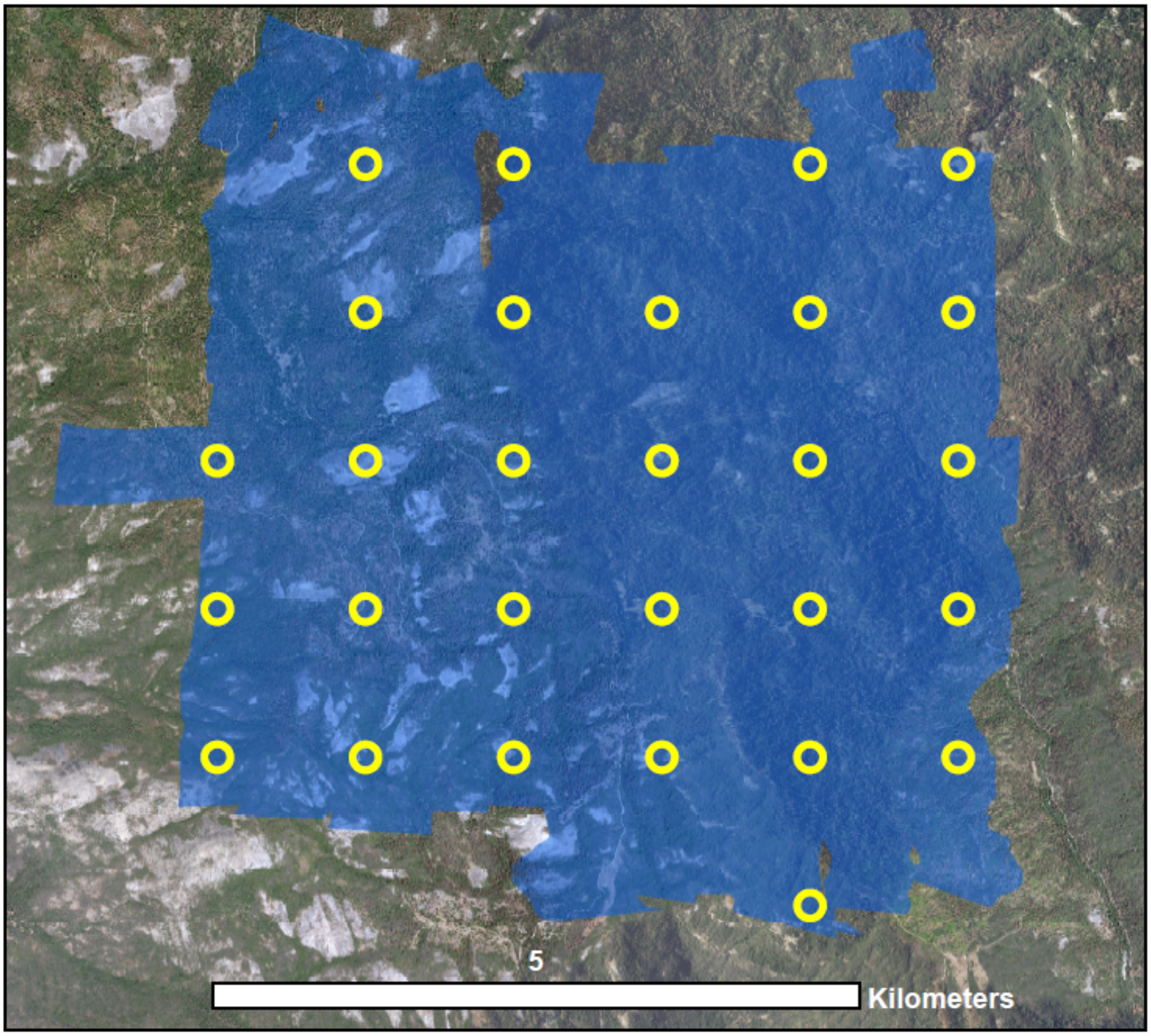

Supplementary Figure 2: Map of validation plots (yellow) used in mortality detection accuracy assessment with lidar acquisition overlay area (blue).

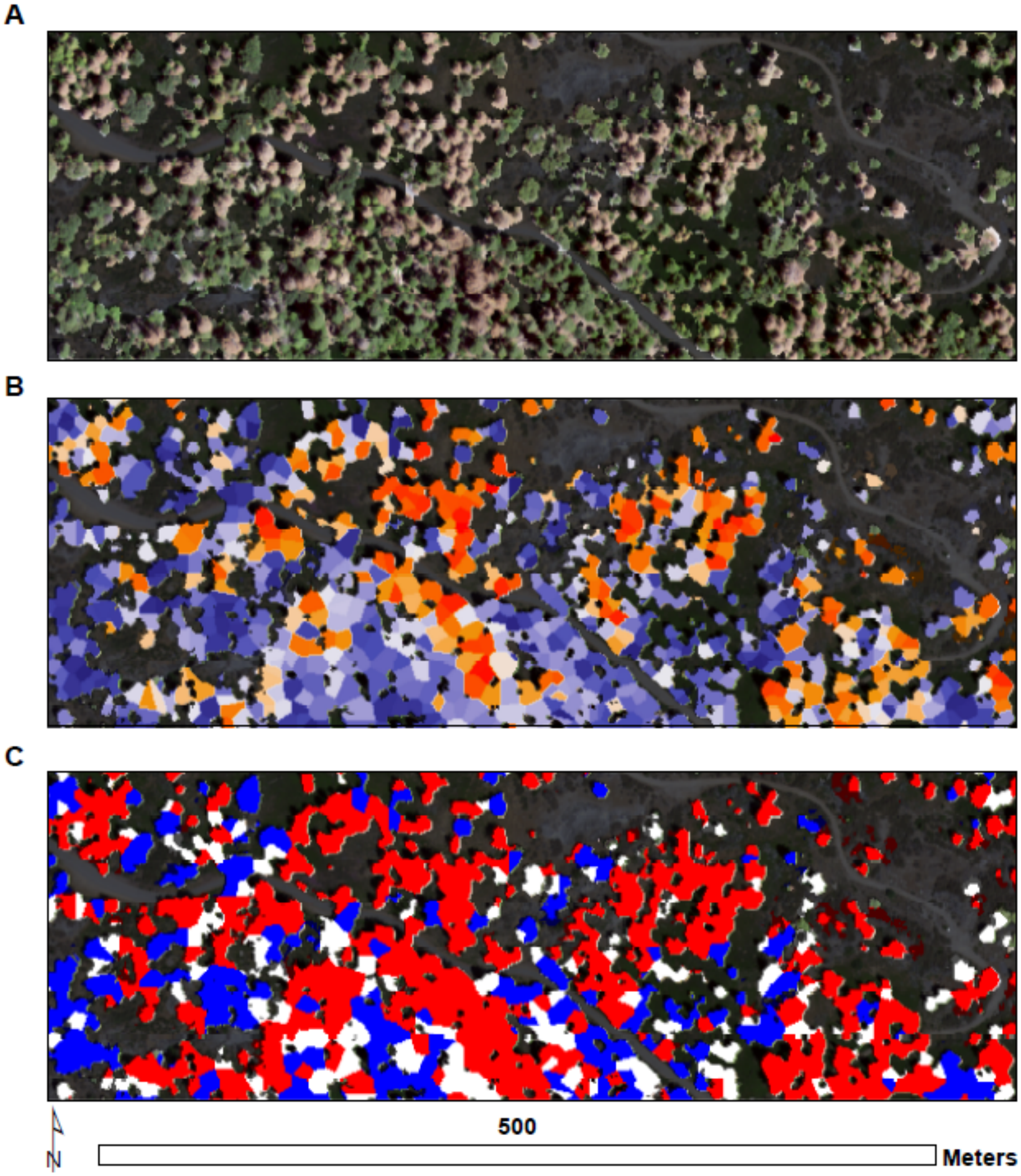

Supplementary Figure 3: Example crown-level classification based on NAIP imagery. Pixels are classified in the multispectral imagery [A] as dead or live and averaged at the crown-level, to estimate the percentage of death [B] for individual tree crowns (0% = blue, 100% = red). [C] We apply a threshold of 37.5% crown death to distinguish living (0 % = blue) and dead (100% = red) trees. Crowns marked in white fall within 5% of the mortality threshold.

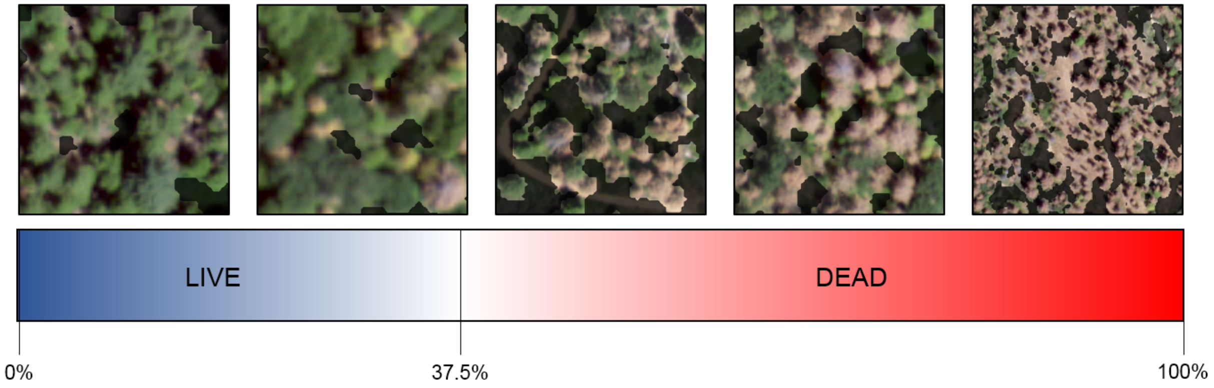

Supplementary Figure 4: Range of crown mortality observed in the study. Crowns with 0% to 37.5% were classified as living and individuals with greater than 37.5% crown mortality were classified as dead.

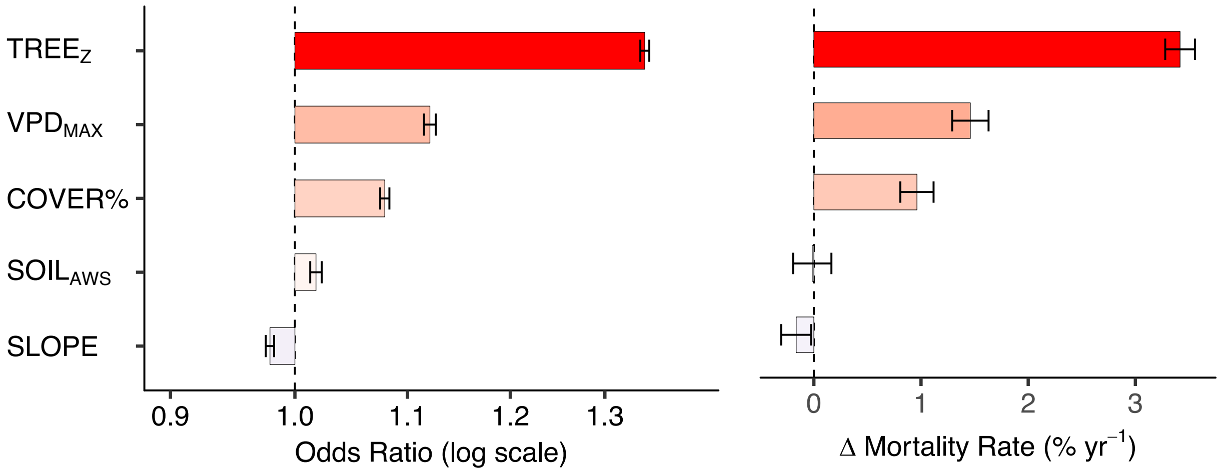

Supplementary Figure 5: Tree height remains the most important risk factor in predicting tree mortality in a reduced model, substituting vapor pressor deficit (VPD_{MAX}) for temperature and precipitation. Including vapor pressure deficit in the model reduces the importance of forest cover and soil texture, but elevates the importance of tree height, increasing the odds ratio from 1.26 to 1.35 and the mortality rate from 2.40 to 3.42 % yr^{-1}. Our findings agree with past work[24] highlighting increased vapor pressor deficit as the single primary climatic driver of tree mortality during drought, since this variable most directly influences stomatal closure and xylem water potential. Error bars represent the 95% confidence in the estimates of the odds ratio and mortality rate.

Supplementary Table 1: Summary statistics for environmental variables included in the analysis of mortality risk. See Supplementary Figure 6 for density distributions.

	$\bar{\mu}$	σ	*min*	*max*
$TREE_Z$ (m)	27.23	11.34	4.18	76.00
VPD_{MAX} (kPa)	1.79	0.37	1.28	2.92
T_{MAX} (^{o}C)	15.8	2.5	11.8	22.2
PPT (mm)	868	65	660	938
AWS (mm)	9.45	6.22	1.96	23.15
COVER (%)	48.43	17.24	0.00	98.11
SLOPE (^{o})	15.80	8.77	0.29	82.71
Elevation (m)	2068	451	897	3078

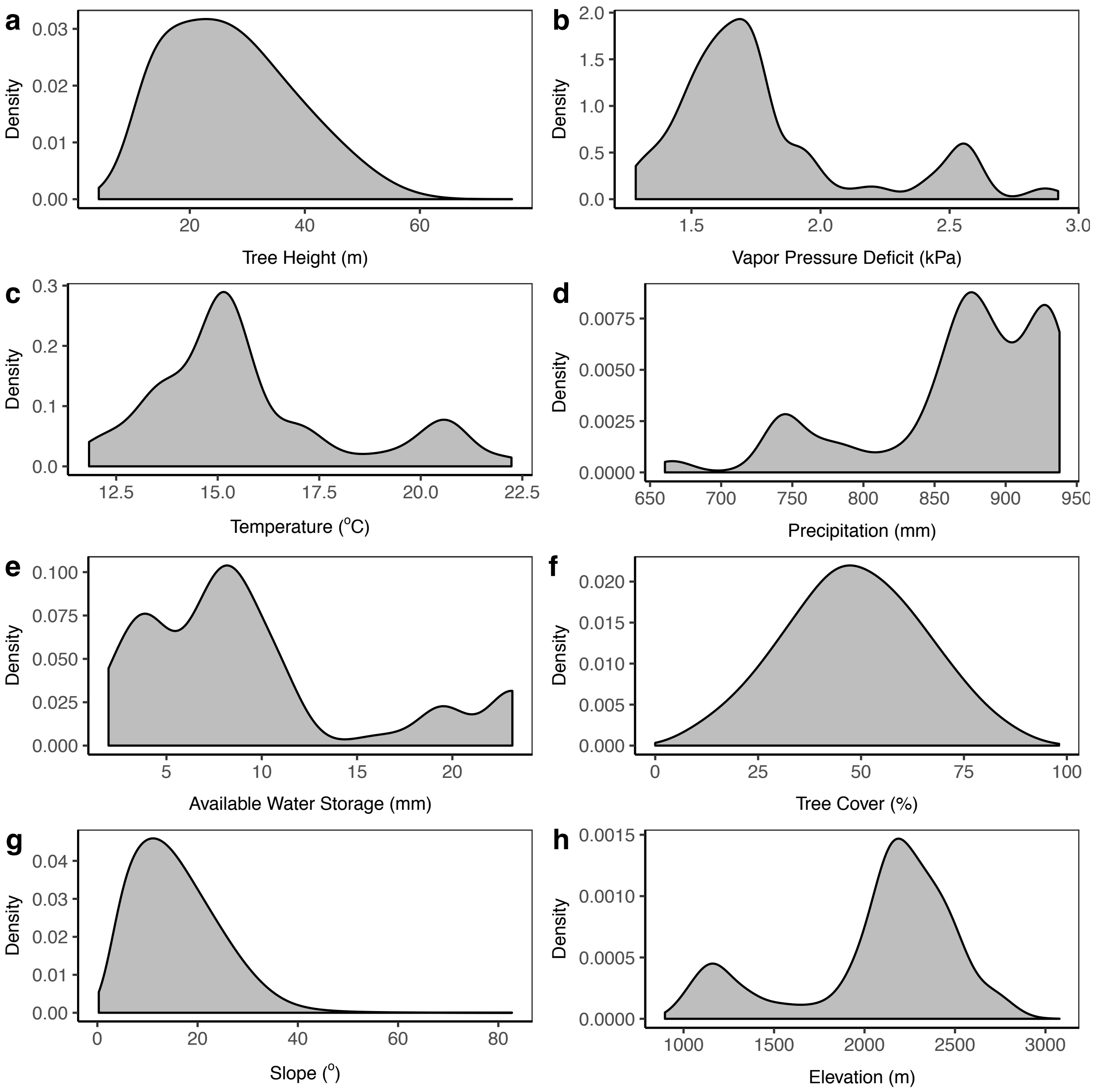

Supplementary Figure 6: Distribution of observed values for all variables tested in the mortality risk analysis. Summary statistics for all variables are given in Supplementary Table 1.

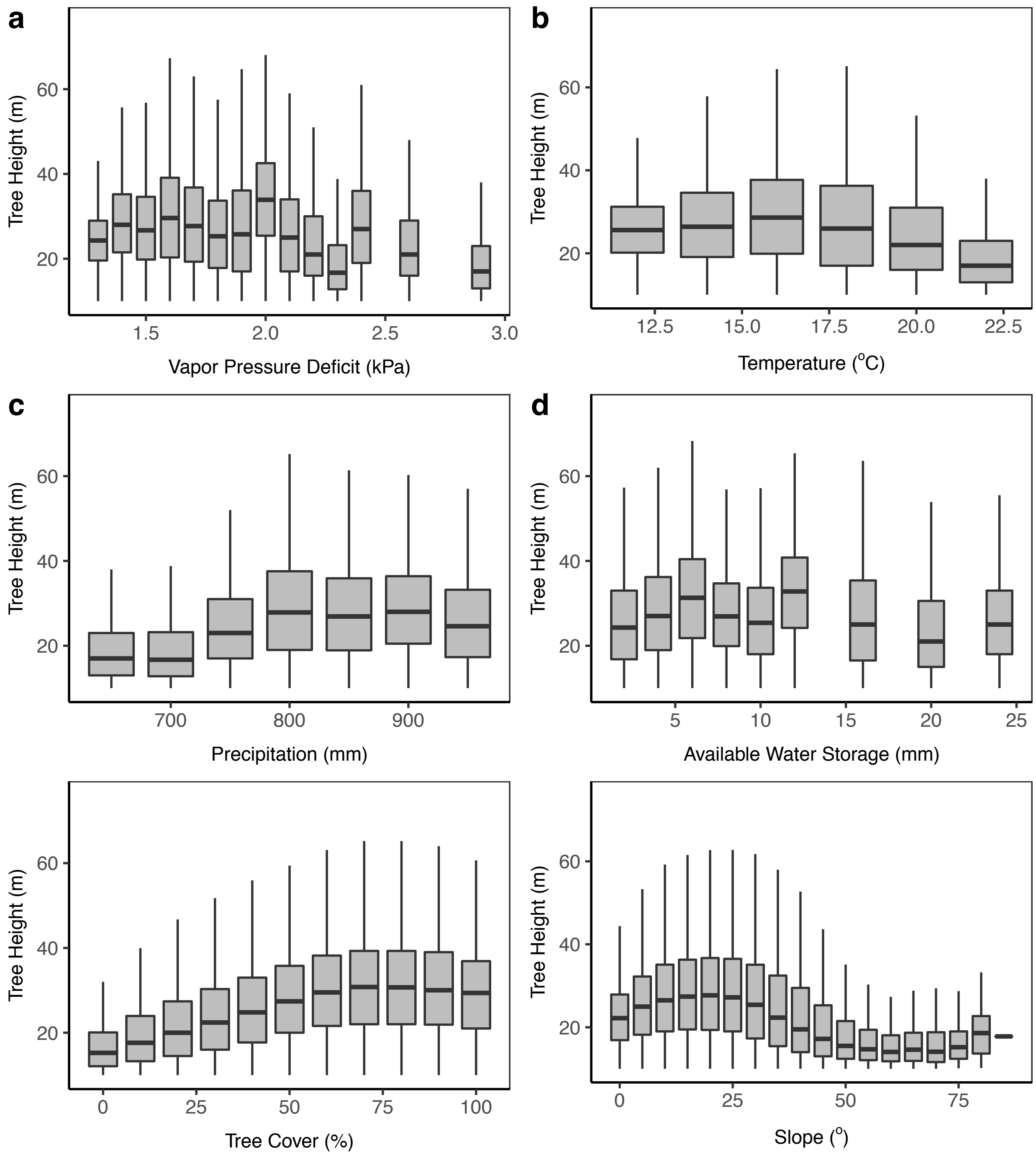

Supplementary Figure 7: Distribution of tree height for all variables tested in the mortality risk analysis.

Supplementary Table 2: Mortality-height intensity model (Fig. 3) coefficients and fit statistics.

	β_1	σ	R^2	p
VPD_{MAX}	0.0938	0.0092	0.88	<0.001
T_{MAX}	0.0762	0.0153	0.73	<0.001
PPT	-0.0038	0.0011	0.46	0.003
AWS	0.0360	0.0111	0.49	0.010
COVER% (0-50%)	0.1481	0.0289	0.83	0.007
COVER% (50-100%)	2.371	0.1985	0.98	0.007
SLOPE	0.0030	0.0012	0.35	0.032

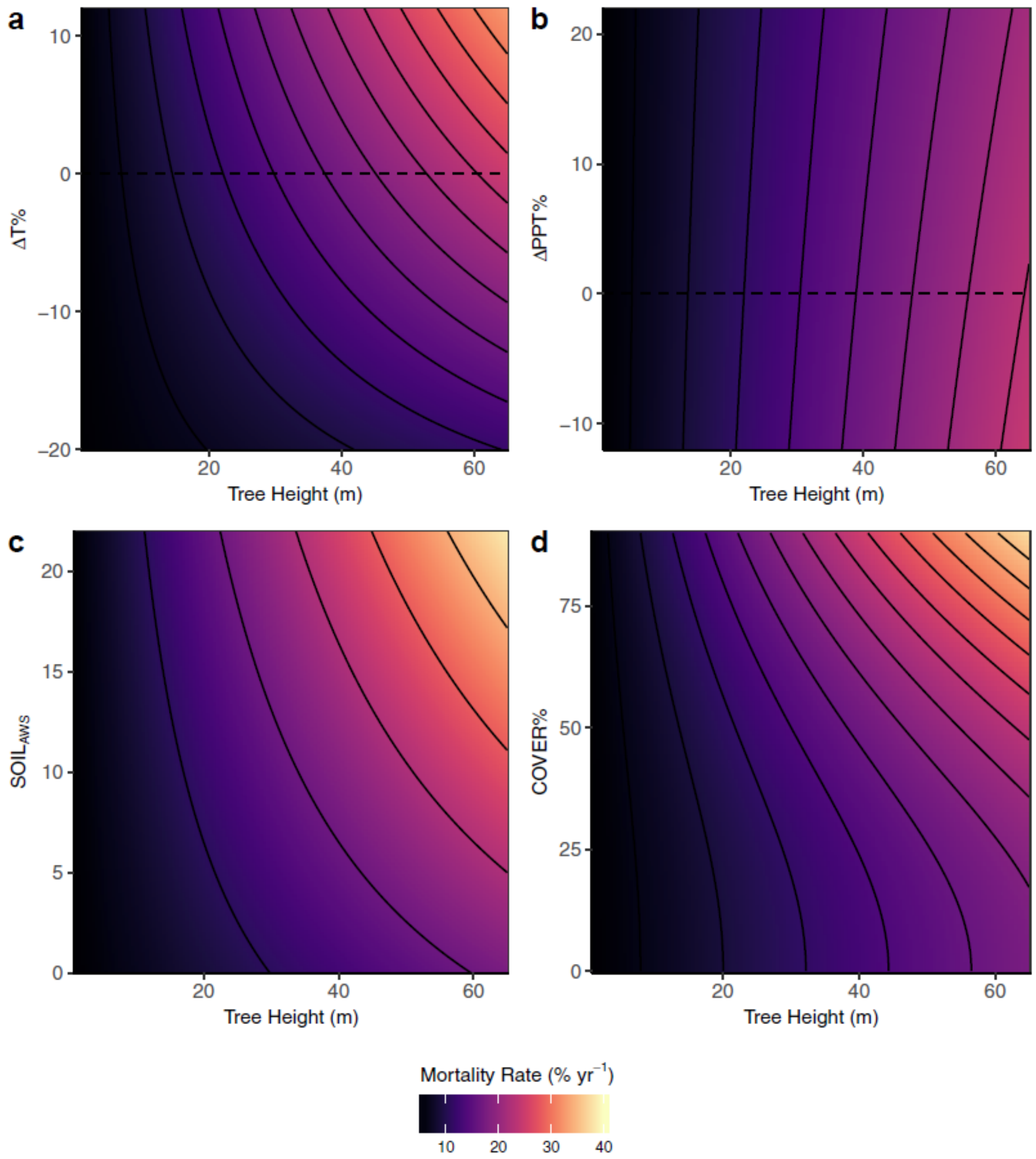

Supplementary Figure 8: Tree height and gradients control tree mortality rate. Dashed line in a-b indicates the normal climate condition (1970-2000). Continuous gradients in mortality rate can parameterize forest growth models and improving estimates of vegetation-climate feedbacks.
