# Supplementary material for: Tree height explains mortality risk during an intense drought
Source: Nat Commun. 2019 Sep 26;10:4385. doi: 10.1038/s41467-019-12380-6 (PMC6763443; doi:10.1038/s41467-019-12380-6)
Supplement: Supplementary file 2 — Description of Additional Supplementary Files [file 41467_2019_12380_MOESM2_ESM.pdf]

Description of Additional Supplementary Files

File Name: Supplementary Movie 1
Description: Full-color LiDAR flight through the high-elevation TEAK site.
