# Supplementary material for: Tree height explains mortality risk during an intense drought
Source: Nat Commun. 2019 Sep 26;10:4385. doi: 10.1038/s41467-019-12380-6 (PMC6763443; doi:10.1038/s41467-019-12380-6)
Supplement: Supplementary file 4 — Reporting Summary [file 41467_2019_12380_MOESM4_ESM.pdf]

nature research

Corresponding author(s): Atticus E.L. Stovall

Last updated by author(s): Aug 6, 2019

Reporting Summary

Nature Research wishes to improve the reproducibility of the work that we publish. This form provides structure for consistency and transparency in reporting. For further information on Nature Research policies, see Authors & Referees and the Editorial Policy Checklist .

Statistics

For all statistical analyses, confirm that the following items are present in the figure legend, table legend, main text, or Methods section.

n/a	Confirmed	
☐	☒	The exact sample size (*n*) for each experimental group/condition, given as a discrete number and unit of measurement
☐	☒	A statement on whether measurements were taken from distinct samples or whether the same sample was measured repeatedly
☐	☒	The statistical test(s) used AND whether they are one- or two-sided<br>*Only common tests should be described solely by name; describe more complex techniques in the Methods section.*
☐	☒	A description of all covariates tested
☐	☒	A description of any assumptions or corrections, such as tests of normality and adjustment for multiple comparisons
☐	☒	A full description of the statistical parameters including central tendency (e.g. means) or other basic estimates (e.g. regression coefficient) AND variation (e.g. standard deviation) or associated estimates of uncertainty (e.g. confidence intervals)
☐	☒	For null hypothesis testing, the test statistic (e.g. *F*, *t*, *r*) with confidence intervals, effect sizes, degrees of freedom and *P* value noted<br>*Give P values as exact values whenever suitable.*
☒	☐	For Bayesian analysis, information on the choice of priors and Markov chain Monte Carlo settings
☒	☐	For hierarchical and complex designs, identification of the appropriate level for tests and full reporting of outcomes
☒	☐	Estimates of effect sizes (e.g. Cohen's *d*, Pearson's *r*), indicating how they were calculated

Our web collection on statistics for biologists contains articles on many of the points above.

Software and code

Policy information about availability of computer code

Data collection	The LiDAR data was analyzed in R using the lidR package. We used the "lastrees_silva" function to delineate detected tree crowns. We linked environmental data to individual tree crowns with the "zonal" and "extract" functions in the raster package. Zonal was used for fine resolution spatial data and used the tree crown as the zonal area. Extract was used for more coarse spatial data and simply extracted the pixel value a the tree coordinates.
Data analysis	All data analysis was carried out in R using the base functions.

For manuscripts utilizing custom algorithms or software that are central to the research but not yet described in published literature, software must be made available to editors/reviewers. We strongly encourage code deposition in a community repository (e.g. GitHub). See the Nature Research guidelines for submitting code & software for further information.

Data

Policy information about availability of data

All manuscripts must include a data availability statement. This statement should provide the following information, where applicable:

- Accession codes, unique identifiers, or web links for publicly available datasets
- A list of figures that have associated raw data
- A description of any restrictions on data availability

The tree-level data generated from the LiDAR data along with associated environmental data are hosted through the following link: https://figshare.com/s/61098e084649e771ff03. All other spatial data are publically available.

Field-specific reporting

Please select the one below that is the best fit for your research. If you are not sure, read the appropriate sections before making your selection.

☐ Life sciences ☐ Behavioural & social sciences ☒ Ecological, evolutionary & environmental sciences

For a reference copy of the document with all sections, see nature.com/documents/nr-reporting-summary-flat.pdf

Ecological, evolutionary & environmental sciences study design

All studies must disclose on these points even when the disclosure is negative.

Study description	Mapping location, size, and mortality of over 1.8 million trees in the Sierra Nevada mountains to determine the primary drivers of tree mortality during extreme drought.
Research sample	We mapped all trees above 5 m within the extent of two LiDAR acquisitions totaling 40,854 ha and continuously spanning over 2000 m of elevation.
Sampling strategy	All trees within the study area were captured and a 5 m canopy height threshold was used to limit the analysis to areas defined as "forest."
Data collection	All spatial data were freely available and collected online from several repositories. The LiDAR data was provided by NEON (National Ecological Observatory Network). The temperature and precipitation during the drought were PRISM datasets. The temperature and precipitation normals (1970-2000) were for WorldClim. The forest cover, slope, and aspect data were derived from the NEON LiDAR data. The NAIP imagery was provided by USDA and acquired from EarthExplorer.
Timing and spatial scale	The study took place between 2009 and 2016. The mortality estimates were based on NAIP imagery acquired in the summer months of 2009, 2010, 2012, 2014, and 2016. The total study area spanned 40,854 ha and corresponded with two LiDAR acquisitions that took place in 2013.
Data exclusions	We also processed the LiDAR data from the San Joaquin Experimental Range (SJER) NEON site. In total, we detected 164,749 trees, but, due to the open canopy and extremely brown ground surface, were unable to identify dead trees with high confidence. We are currently exploring other techniques to make use of these data and better understand mortality trends in dry, low elevation sites.
Reproducibility	We have simplified the analysis conducted in R into a pipeline of processing scripts that produce the final tree-level dataset. We will make the analysis publicly available on GitHub prior to publication of the manuscript.
Randomization	Randomization was unnecessary in our analysis because we fully sampled all trees within the study area. We controlled for covariates in our analysis of variable odds ratio by using multiple regression analysis on all standardized variables to determine the relative variable effect size. We tested for collinearity with variance inflation factors, ensuring all VIFs were less than 2.
Blinding	Blinding was not relevant to this study because we relied on an automated sampling method.

Did the study involve field work? ☐ Yes ☒ No

Reporting for specific materials, systems and methods

We require information from authors about some types of materials, experimental systems and methods used in many studies. Here, indicate whether each material, system or method listed is relevant to your study. If you are not sure if a list item applies to your research, read the appropriate section before selecting a response.

Materials & experimental systems

n/a	Involved in the study
☒	☐ Antibodies
☒	☐ Eukaryotic cell lines
☒	☐ Palaeontology
☒	☐ Animals and other organisms
☒	☐ Human research participants
☒	☐ Clinical data

Methods

n/a	Involved in the study
☒	☐ ChIP-seq
☒	☐ Flow cytometry
☒	☐ MRI-based neuroimaging
